# Severe Refractory Hypercalcemia as the Initial Presentation of Sarcoidosis With Acute Kidney Injury Requiring Hemodialysis

**DOI:** 10.7759/cureus.109656

**Published:** 2026-05-25

**Authors:** Shalima Sabu, Jishnu Mohan

**Affiliations:** 1 Internal Medicine, Ananthapuri Hospitals and Research Institute, Thiruvananthapuram, IND; 2 Nephrology, Ananthapuri Hospitals and Research Institute, Thiruvananthapuram, IND

**Keywords:** acute kidney injury, denosumab and sarcoidosis, extra pulmonary manifestations of sarcoidosis, granulomatous disease, hemodialysis, noncaseating granulomas, refractory hypercalcemia, vitamin d activation

## Abstract

Sarcoidosis is a multisystem granulomatous disorder that most commonly involves the lungs and intrathoracic lymph nodes. Hypercalcemia is a recognized but uncommon manifestation of sarcoidosis and is typically mild; however, severe hypercalcemia as the initial presentation is rare and may pose a diagnostic challenge. We report a 66-year-old female patient with multiple comorbidities, including stage 4 chronic kidney disease, who presented with fatigue, decreased appetite, vomiting, and significant weight loss. Laboratory findings demonstrated severe hypercalcemia (15.7 mg/dL) with acute worsening of renal function. Parathyroid hormone (PTH) levels were inappropriately normal in this setting. Neck ultrasonography suggested a possible parathyroid lesion; however, technetium-99m sestamibi scintigraphy showed no focal uptake, excluding a functional adenoma. Further workup excluded malignancy and vitamin D intoxication. Elevated 1,25-dihydroxyvitamin D levels suggested extrarenal activation. Positron emission tomography-computed tomography (PET/CT) revealed widespread lymphadenopathy, and mediastinal lymph node biopsy confirmed noncaseating granulomas, establishing the diagnosis of sarcoidosis. The patient developed refractory hypercalcemia requiring aggressive medical therapy, denosumab, and one session of hemodialysis. Initiation of corticosteroid therapy resulted in rapid clinical and biochemical improvement. This case highlights severe vitamin D-mediated hypercalcemia as an uncommon presentation of sarcoidosis with acute kidney injury. Cases requiring both denosumab and hemodialysis are rarely reported. A systematic approach to evaluation is essential, particularly in distinguishing PTH-dependent from independent causes. Sarcoidosis should be considered in unexplained severe hypercalcemia, even in the absence of pulmonary manifestations, as early recognition and corticosteroid therapy can be highly effective.

## Introduction

Hypercalcemia is a potentially life-threatening electrolyte disturbance, affecting approximately one percent of the population [1]. It is classified based on severity, with severe hypercalcemia associated with gastrointestinal symptoms, dehydration, neurocognitive impairment, cardiac arrhythmias, and acute kidney injury [1,2]. The most common causes are primary hyperparathyroidism and malignancy, accounting for nearly 90% of cases, while less common etiologies include granulomatous diseases such as sarcoidosis and tuberculosis, medications, and endocrine disorders [2,3]. 

Sarcoidosis is a multisystem granulomatous disorder of unknown etiology characterized by the formation of noncaseating granulomas. It most commonly affects the lungs and intrathoracic lymph nodes, but extrapulmonary involvement is seen in a substantial proportion of patients, including the skin, eyes, liver, heart, nervous system, and kidneys [4]. Renal involvement may result from hypercalcemia-induced nephrocalcinosis, nephrolithiasis, or granulomatous interstitial nephritis, all of which can contribute to acute or chronic kidney dysfunction [4]. 

Disturbances in calcium metabolism are well-recognized in sarcoidosis and are primarily mediated by dysregulated vitamin D metabolism. Activated macrophages within granulomas express 1α-hydroxylase, leading to increased extrarenal conversion of 25-hydroxyvitamin D to 1,25-dihydroxyvitamin D (calcitriol), independent of normal physiological feedback mechanisms [2,5]. This results in increased intestinal calcium absorption and subsequent hypercalcemia, which may be further exacerbated in patients with renal impairment due to reduced calcium excretion. 

Although hypercalcemia occurs in approximately 10-20% of patients with sarcoidosis, it is usually mild and asymptomatic. Severe hypercalcemia as the initial manifestation is uncommon and may present without classical pulmonary findings, posing a diagnostic challenge [5,6]. We report a case of sarcoidosis presenting with severe refractory hypercalcemia and acute kidney injury requiring hemodialysis, highlighting the diagnostic challenges and the importance of a systematic approach.

## Case presentation

A 66-year-old female patient presented with fatigue and decreased appetite for one week, along with two episodes of vomiting over one day. She also reported significant unintentional weight loss of approximately 18 kg over the preceding six months. She denied fever, cough, dyspnea, chest pain, bone pain, constipation, polyuria, polydipsia, decreased urine output, or neuropsychiatric symptoms. Her family and social history were unremarkable. Initial evaluation at a local clinic revealed severe hypercalcemia (15.7 mg/dL) and elevated serum creatinine (4.1 mg/dL) compared to a baseline of 2.5 mg/dL, prompting referral for further management. 

Her past medical history was significant for type 2 diabetes mellitus, systemic hypertension, dyslipidemia, stage 4 chronic kidney disease, hypothyroidism, myxomatous mitral valve disease, frequent ventricular premature complexes, right renal angiomyolipoma, and prior spontaneous retinal detachment. Her home medications included metoprolol, prazosin, telmisartan-amlodipine, rosuvastatin, metformin, moxonidine, levothyroxine, and methylcobalamin. 

On examination, she was conscious, oriented, and afebrile, with features of dehydration, including dry oral mucosa, reduced skin turgor, and delayed capillary refill. Mild bilateral pitting pedal edema was noted. Her pulse rate was 90 beats per minute, blood pressure was 180/90 mmHg, respiratory rate was 18 breaths per minute, and oxygen saturation was 99% on room air. Systemic examination was otherwise unremarkable. Initial laboratory investigations are summarized in Table 1.

**Table 1:** Initial laboratory investigations at presentation ESR: erythrocyte sedimentation rate; SGOT (AST): serum glutamic-oxaloacetic transaminase (aspartate aminotransferase); SGPT (ALT): serum glutamic-pyruvic transaminase (alanine aminotransferase); ALP: alkaline phosphatase; RBC: red blood cells; HPF: high-power field Baseline hematological, biochemical, and urinalysis parameters at presentation demonstrating severe hypercalcemia and acute kidney injury

Parameter	Result	Reference range
Hemoglobin	10.7 g/dL	12.0-16.0 g/dL
Total leukocyte count	7.01 × 10³/µL	4.0-11.0 × 10³/µL
Neutrophil	68%	40-75%
Lymphocyte	23%	20-45%
Platelet	2.13 × 10⁵/µL	1.5-4.5 × 10⁵/µL
ESR	36 mm/hr	<20 mm/hr
Total bilirubin	0.4 mg/dL	0.4-1.2 mg/dL
SGOT (AST)	17 U/L	5-40 U/L
SGPT (ALT)	14 U/L	≤35 U/L
ALP	79 U/L	5-120 U/L
Total proteins	7.7 g/dL	6.3-8.2 g/dL
Albumin	4.2 g/dL	3.5-5 g/dL
Globulins	3.5 g/dL	2-3.6 g/dL
Urea	105 mg/dL	15-45 mg/dL
Creatinine	4.3 mg/dL	0.6-1.3 mg/dL
Sodium	136 mmol/L	135-145 mmol/L
Potassium	3.6 mmol/L	3.5-5.1 mmol/L
25-hydroxyvitamin D	70.9 ng/mL	Sufficient (25-80 ng/mL)
Phosphorus	5.1 mg/dL	2.4-4.7 mg/dL
Uric acid	9 mg/dL	2.5-6.2 mg/dL
Calcium	15.3 mg/dL	8.4-10.2 mg/dL
Magnesium	1.5 mg/dL	1.7–2.7 mg/dL
Urine pH	5.5	5–8
Urine sugar	Nil	Nil
Urine ketones	Negative	Negative
Urine albumin	1+	Nil
Urine nitrites	Negative	Negative
Urine bilirubin	Negative	Negative
Urobilinogen	Normal	Normal
Urine pus cells	4 cells/HPF	2-3 cells/HPF
Urine epithelial cells	1 cell/HPF	2-3 cells/HPF
Urine RBC	<1 cell/HPF	Nil
Urine crystals	Nil	Nil
Urine casts	Nil	Nil
Random blood glucose	97 mg/dL	<140 mg/dL

**Figure 1:**
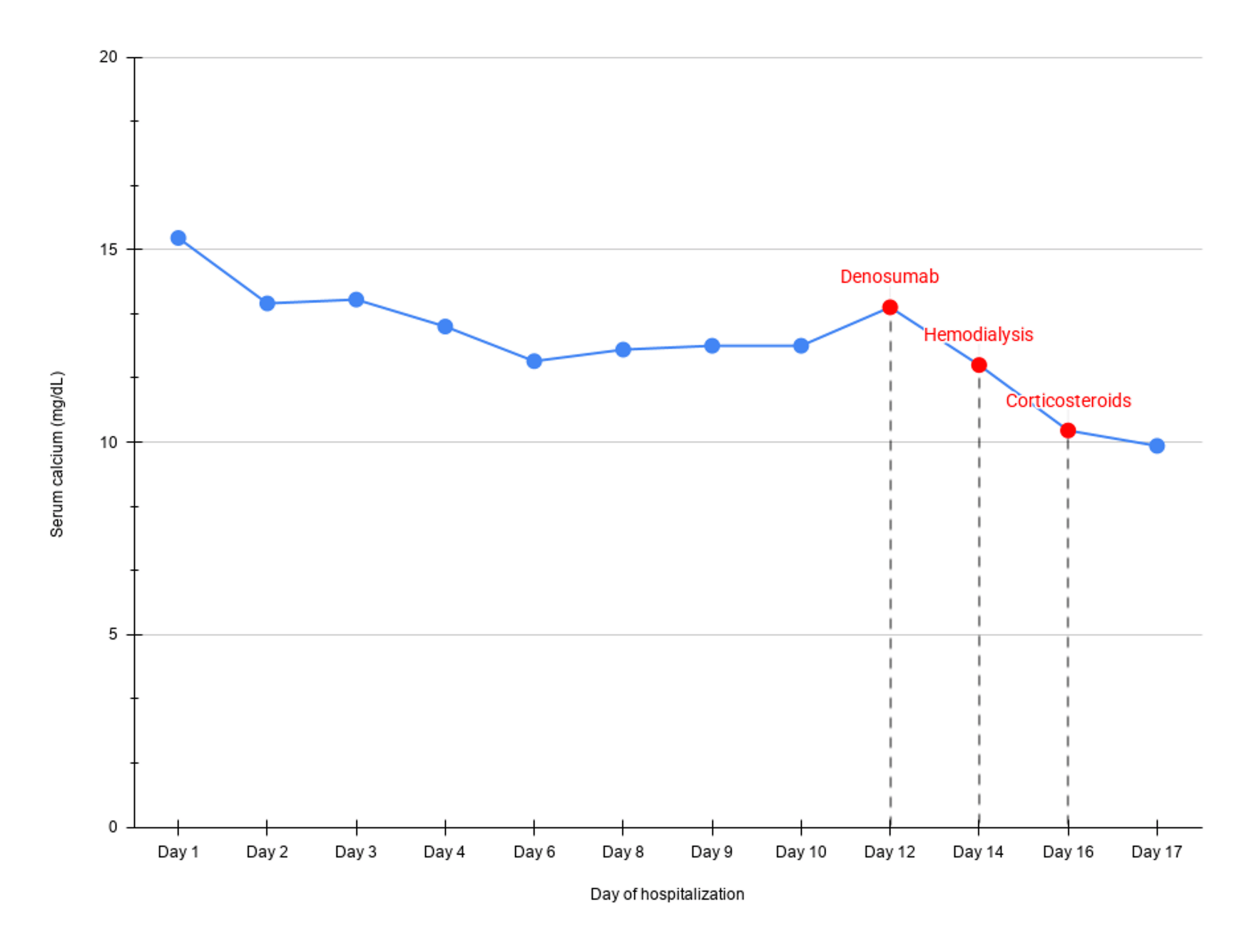
Trend of serum calcium levels during hospitalization Serial serum calcium measurements during hospitalization demonstrating persistent hypercalcemia despite initial therapy, with subsequent decline following escalation of treatment with denosumab, hemodialysis, and subsequent corticosteroid therapy

Laboratory investigations revealed severe hypercalcemia and acute kidney injury. The patient was admitted to the medical intensive care unit and managed with aggressive intravenous hydration, calcitonin, and loop diuretics. Persistent hypercalcemia was noted on serial monitoring (Figure 1).

To differentiate parathyroid hormone (PTH)-dependent from PTH-independent causes, intact PTH was measured and found to be 30 pg/mL, which was inappropriately normal in the setting of severe hypercalcemia, suggesting a PTH-independent etiology. Neck ultrasonography suggested a possible parathyroid lesion; however, technetium-99m sestamibi scintigraphy showed no focal uptake, excluding a functional adenoma. 

Serum 25-hydroxyvitamin D levels were normal, excluding vitamin D intoxication. Twenty-four-hour urinary calcium excretion was elevated, ruling out familial hypocalciuric hypercalcemia. Given her age and significant weight loss, malignancy was strongly considered. Further evaluation is summarized in Table 2.

**Table 2:** Evaluation for malignancy and plasma cell dyscrasia ACE: angiotensin-converting enzyme; CA 125: cancer antigen 125; Ig: immunoglobulin

Parameter	Result	Reference/expected finding
Comprehensive myeloma panel	-	-
Serum protein capillary electrophoresis	-	-
Total protein	Within normal limits	Within normal limits
Albumin fraction	Decreased	Within normal limits
Alpha-1 globulin fraction	Increased	Within normal limits
Alpha-2 globulin fraction	Within normal limits	Within normal limits
Beta-1 globulin fraction	Within normal limits	Within normal limits
Beta-2 globulin fraction	Within normal limits	Within normal limits
Gamma globulin fraction	Increased	Within normal limits
M-spike	Not detected	Not detected
Immunofixation electrophoresis	-	-
Myeloma peak/band	Not detected	Not detected
IgG peak	Not detected	Not detected
IgA peak	Not detected	Not detected
IgM peak	Not detected	Not detected
Kappa fraction	Not detected	Not detected
Lambda fraction	Not detected	Not detected
Serum free light chain assay	-	-
Kappa free light chain	104.78 mg/L	3.3-19.4 mg/L
Lambda free light chain	115.73 mg/L	5.71-26.3 mg/L
Kappa/lambda ratio	0.910	0.26-1.65
Total IgA	2.72 g/L	0.9-4.1 g/L
Total IgG	15.80 g/L	6-15.6 g/L
Total IgM	1.40 g/L	0.3-3.6 g/L
Beta-2 microglobulin	9264 ng/mL	609.0-2366.0 ng/mL
Tumor marker evaluation	-	-
CA 125	8.00 U/mL	≤35 U/mL
Sarcoidosis-related evaluation	-	-
ACE	32.5 U/L	8-52 U/L

Tumor markers were within normal limits. Evaluation for plasma cell dyscrasia showed no monoclonal band on serum protein electrophoresis, with a normal serum free light chain ratio; however, beta-2 microglobulin was elevated at 9264 ng/mL. Serum angiotensin-converting enzyme levels were within the normal range; however, normal ACE levels do not exclude sarcoidosis due to limited sensitivity. 

Further evaluation revealed elevated 1,25-dihydroxyvitamin D levels, suggesting extrarenal activation and raising suspicion for granulomatous diseases such as sarcoidosis or tuberculosis. Mantoux test, acid-fast bacilli smear, and *Mycobacterium tuberculosis* testing were negative. 

**Figure 2:**
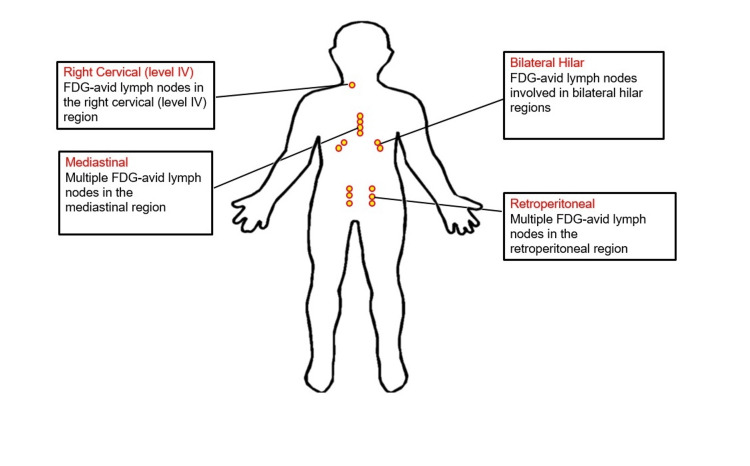
Schematic representation of FDG PET/CT findings demonstrating FDG-avid lymphadenopathy involving the right cervical (level IV), mediastinal, bilateral hilar, and retroperitoneal regions FDG PET/CT: fluorodeoxyglucose positron emission tomography-computed tomography

Fluorodeoxyglucose positron emission tomography-computed tomography (FDG PET/CT) demonstrated multiple FDG-avid lymph nodes involving the right cervical (level IV), mediastinal, bilateral hilar, and retroperitoneal regions, suggestive of a systemic inflammatory or neoplastic process, warranting tissue diagnosis (Figure 2). No focal hepatic or skeletal lesions were identified.

Due to refractory hypercalcemia with worsening acute-on-chronic kidney injury, denosumab was administered because of its favorable safety profile in advanced renal dysfunction compared with bisphosphonates. As hypercalcemia persisted and denosumab has a delayed onset of action, one session of hemodialysis was performed as adjunctive therapy to facilitate calcium removal and renal stabilization. 

Given the overall clinical picture, a granulomatous etiology was strongly suspected. Endobronchial ultrasound-guided biopsy with mediastinal cryobiopsy was performed. Histopathological examination revealed lymphoid tissue with multiple confluent and discrete noncaseating granulomas composed of epithelioid histiocytes and multinucleated giant cells, without evidence of malignancy, consistent with granulomatous lymphadenitis. 

Corticosteroid therapy was initiated, resulting in significant clinical improvement, with normalization of serum calcium levels and gradual recovery of renal function. Blood pressure and metabolic parameters improved, and the patient was discharged in stable condition with advice for nephrology follow-up.

## Discussion

Severe hypercalcemia as the initial manifestation of sarcoidosis is uncommon and clinically significant, particularly when associated with acute kidney injury. Although disturbances in calcium metabolism occur in approximately 10-20% of patients with sarcoidosis, they are typically mild and asymptomatic, and presentations requiring renal replacement therapy are rarely reported in the literature [5,6].

The evaluation of hypercalcemia requires a systematic and stepwise approach, beginning with differentiation between PTH-dependent and PTH-independent mechanisms. In this case, the PTH level was inappropriately normal in the context of severe hypercalcemia, indicating a PTH-independent etiology. This biochemical profile, combined with normal 25-hydroxyvitamin D and elevated 1,25-dihydroxyvitamin D levels, strongly suggested extrarenal calcitriol production, a hallmark of granulomatous diseases such as sarcoidosis [2,5]. Activated macrophages within granulomas express 1-α-hydroxylase independent of normal feedback regulation, resulting in increased intestinal calcium absorption and subsequent hypercalcemia.

The differential diagnosis of PTH-independent hypercalcemia includes malignancy, vitamin D intoxication, granulomatous diseases, medications, and endocrine disorders. In this patient, malignancy was initially a major diagnostic consideration given advanced age and significant weight loss. However, the absence of monoclonal protein on serum protein electrophoresis, a normal serum free light chain ratio, and the lack of focal lesions on imaging made this diagnosis unlikely. Tuberculosis was also excluded based on negative microbiological testing and the absence of caseating granulomas. Definitive diagnosis was established by histopathological identification of non-caseating granulomas, confirming sarcoidosis.

Recent literature increasingly recognizes that sarcoidosis may present with isolated extrapulmonary manifestations, including hypercalcemia and renal dysfunction, even in the absence of pulmonary involvement. A recent case report described renal sarcoidosis presenting with acute kidney injury and persistent hypercalcemia without respiratory symptoms, underscoring the diagnostic complexity of such atypical presentations [7]. Similarly, other contemporary reports have demonstrated that severe hypercalcemia may be the dominant presenting feature, often prompting extensive evaluation for malignancy before a granulomatous etiology is identified [8].

Management of severe hypercalcemia requires both rapid correction of serum calcium levels and targeted treatment of the underlying cause. Initial therapy includes aggressive intravenous hydration and calcitonin, followed by antiresorptive agents for sustained control. Although bisphosphonates are commonly used, their use may be limited in patients with advanced renal dysfunction due to the risk of nephrotoxicity. In such cases, denosumab represents an effective alternative for patients with renal dysfunction. Denosumab, a monoclonal antibody targeting receptor activator of nuclear factor kappa-B ligand (RANKL), inhibits osteoclast-mediated bone resorption and reduces calcium mobilization from bone [9,10].

Emerging evidence supports the use of denosumab in sarcoidosis-related hypercalcemia, particularly in patients with renal impairment or refractory disease [11]. Denosumab has a delayed onset of action, with initial calcium-lowering effects typically observed within 24-72 hours and maximal effects occurring several days after administration, which may explain the absence of an immediate decline in serum calcium following treatment in this case. While most reported cases demonstrate resolution with medical therapy alone, the present case is notable for requiring both denosumab and hemodialysis, reflecting a more severe and refractory clinical course. Such combined management has been infrequently described, highlighting the clinical severity and therapeutic challenges in this subset of patients.

Glucocorticoids remain the cornerstone of therapy in sarcoidosis-related hypercalcemia, as they suppress macrophage-driven calcitriol production and decrease intestinal calcium absorption [12]. Clinical improvement is often rapid following initiation, as observed in this patient. Early recognition of the underlying mechanism is therefore critical, as it directly influences management decisions and helps avoid unnecessary or potentially harmful interventions.

This case underscores several key clinical insights. First, sarcoidosis should be considered in the differential diagnosis of severe hypercalcemia, even in the absence of pulmonary manifestations. Second, measurement of 1,25-dihydroxyvitamin D is crucial in identifying vitamin D-mediated hypercalcemia and guiding diagnosis. Third, denosumab is a valuable therapeutic option in patients with renal impairment or refractory hypercalcemia. Finally, severe cases may require escalation to hemodialysis, emphasizing the importance of early diagnosis and timely intervention to prevent complications.

The combination of severe hypercalcemia, advanced renal dysfunction, and the requirement for both denosumab and hemodialysis represents a rare clinical scenario that highlights the importance of early recognition and individualized management in atypical presentations of sarcoidosis.

## Conclusions

This case highlights severe refractory hypercalcemia as an uncommon initial presentation of sarcoidosis occurring in the absence of classic pulmonary manifestations and complicated by acute kidney injury requiring hemodialysis. It underscores the importance of a systematic diagnostic approach to hypercalcemia, particularly in distinguishing PTH-dependent from independent causes and considering granulomatous diseases in the differential diagnosis. Elevated 1,25-dihydroxyvitamin D levels and histopathological identification of noncaseating granulomas were pivotal in establishing the diagnosis. Early recognition and timely initiation of corticosteroid therapy are essential, as they can result in rapid clinical and biochemical improvement. This case emphasizes the need for heightened clinical suspicion for atypical presentations of sarcoidosis to facilitate prompt diagnosis and appropriate management.

## References

[1] Walker MD, Shane E (2022). Hypercalcemia: a review. JAMA.

[2] Tonon CR, Silva TA, Pereira FW (2022). A review of current clinical concepts in the pathophysiology, etiology, diagnosis, and management of hypercalcemia. Med Sci Monit.

[3] Potts JT Jr, Jüppner H (2022). Disorders of the parathyroid gland and calcium homeostasis. Harrison's Principles of Internal Medicine.

[4] Sève P, Pacheco Y, Durupt F (2021). Sarcoidosis: a clinical overview from symptoms to diagnosis. Cells.

[5] Gwadera Ł, Białas AJ, Iwański MA, Górski P, Piotrowski WJ (2019). Sarcoidosis and calcium homeostasis disturbances-do we know where we stand?. Chron Respir Dis.

[6] Xu D, Tao X, Fan Y, Teng Y (2025). Sarcoidosis: molecular mechanisms and therapeutic strategies. Mol Biomed.

[7] Khan MJ, Akbar MA, Ahmad T, Zahid A, Faisal F (2026). Renal sarcoidosis presenting as acute kidney injury with persistent hypercalcemia in the absence of respiratory symptoms: a report of a rare case. Cureus.

[8] Wang Y, Ott M, Ekdahl R, Costa A (2026). Severe hypercalcemia and acute kidney injury as initial manifestations of multisystem sarcoidosis in an adolescent with type 1 diabetes: a case report. Cureus.

[9] El-Hajj Fuleihan G, Clines GA, Hu MI (2023). Treatment of hypercalcemia of malignancy in adults: an endocrine society clinical practice guideline. J Clin Endocrinol Metab.

[10] Fizazi K, Carducci M, Smith M (2011). Denosumab versus zoledronic acid for treatment of bone metastases in men with castration-resistant prostate cancer: a randomised, double-blind study. Lancet.

[11] Fujita N, Ono Y, Hashimoto K (2024). Efficacy of denosumab in the treatment of hypercalcemic renal dysfunction in sarcoidosis: a case report. Osteoporos Int.

[12] Baughman RP, Valeyre D, Korsten P (2021). ERS clinical practice guidelines on treatment of sarcoidosis. Eur Respir J.

